# Supplementary material for: Blood genome expression profiles in infants with congenital cytomegalovirus infection
Source: Nat Commun. 2020 Jul 15;11:3548. doi: 10.1038/s41467-020-17178-5 (PMC7363904; doi:10.1038/s41467-020-17178-5)
Supplement: Supplementary file 1 — Supplementary Information [file 41467_2020_17178_MOESM1_ESM.pdf]

Supplemental Tables and Figures

Blood Genome Expression Profiles in Infants with Congenital Cytomegalovirus Infection

Ouellette et al.

Supplementary Table 1: Demographic characteristics of healthy controls included and excluded from downstream analyses.

	HC included (n=10)	HC excluded (n=11)	p-value
Age (days)	11 (7-33)	21 (1-50)	> 0.99
Gestational age (weeks)	38 (35-40)	40 (39-40)	0.062
Sex (M:F)	7:3	5:6	0.39
Race<br>White<br>Black/other	 8<br>2	 7<br>4	0.64

HC: healthy controls, M; males, F: females. Statistical analyses were performed using Mann Whitney U test for non-parametric continuous variables and data reported as median, 25%-75% interquartile ranges, whereas the Fisher's exac or Chi-square test were used for categorical data. p values were considered significant when $p<0.05$ using two-tailed tests.

Supplementary Table 2: Characteristics of infants with symptomatic cCMV infection included and excluded from downstream analyses.

	Symptomatic CMV Included (n=49)	Symptomatic CMV Excluded (n=6)	P value
Demographic Information			
Sex, males, n (%)	30 (61%)	2 (33%)	>0.99
Gestational age (weeks)	39 (38-40)	37.5 (36-39)	0.10
Birth weight (grams)	3,035 (2,457-3,278)	2,968 (2,199-3,190)	0.52
Birth length (cm)	48 (46-49)	47 (42.5-48.5)	0.23
Head circumference (cm)	33.4 (32-34.4)	33 (29.5-33.5)	0.22
Age at sample collection (days)	17 (11-22)	17 (9.5-23)	0.99
Physical Exam findings, n (%)			
Rash	16 (33%)	2 (33%)	>0.99
Splenomegaly	12 (24%)	3 (50%)	0.66
Hepatomegaly	11 (22%)	2 (33%)	0.62
SGA	10 (20%)	0	0.58
Microcephaly	8 (16%)	2 (33%)	0.30
IUGR	5 (10%)	1 (17%)	0.51
Laboratory results			
WBC (cells/mm^3)	10,735 (8,355-13,133)	13,645 (10,830-16,528)	0.20
Hemoglobin (g/dL)	13.6 (11.3-17.2)	17 (13.7-18.9)	0.14
Hematocrit (%)	40.1 (33.4-48.4)	49.6 (38.9-55)	0.16
Platelet count (/mm^3)	257,000 (94,250-391,500)	290,000 (131,000-442,250)	0.80
ALT (U/L)	20 (14-31)	25 (15.5-63.5)	0.49
Direct bilirubin (mg/dL)	0.3 (0.2-0.53)	0.4 (0.2-3)	0.63
Audiologic findings, n (%)			
Abnormal ABR at any time	22 (45%)	2 (33%)	0.69
Abnormal initial ABR	9 (18%)	0	0.57
Abnormal follow up ABR	13 (32%)	2 (33%)	0.66

CMV, cytomegalovirus; SGA, small for gestational age; IUGR, intrauterine growth restriction; WBC, white blood cell; ALT, alanine aminotransferase; CT, computed tomography; MRI, magnetic resonance imaging; ABR, auditory brainstem response. Statistical analyses were performed using Mann-Whitney U test for non-parametric continuous variables and data reported as median, 25%-75% interquartile ranges, whereas the Fisher's exact or Chi-square test were used for categorical data. p values were considered significant when $p<0.05$ using two-tailed tests.

Supplementary Table 3: Demographic and clinical characteristics of healthy controls and infants with symptomatic and asymptomatic cCMV infection included in the discovery cohort

	Healthy Controls n=10	Symptomatic Congenital CMV n=25	Asymptomatic Congenital CMV n=16	p-value
Age at enrollment (days)	11 [7-33]	17 [11-20]	15.5 [10.5-21]	0.73
Gestational age (weeks)	38 [35-40]	39 [38-40]	39 [39-40]	0.12
Sex, males, n (%)	7 (70%)	13 (52%)	8 (50%)	0.56
Race, n,<br>White<br>Black or other	 8<br>2	 20<br>5	 15<br>1	0.46

Statistical analyses were performed using Kruskal-Wallis test for non-parametric continuous variables and data reported as median, 25%-75% interquartile ranges, whereas the Fisher's exact or Chi-square test were used for categorical data. p values were considered significant when $p<0.05$ using two-tailed tests.

Supplementary Table 4: Top 10 upregulated and downregulated genes identified in the discovery sets of infants with symptomatic or asymptomatic cCMV infection

Symptomatic cCMV biosignature (2,592 genes)				Asymptomatic cCMV biosignature (3,324 genes)			
Top 10 upregulated	Fold change	Top 10 downregulated	Fold change	Top 10 upregulated	Fold change	Top 10 downregulated	Fold change
IFI44L	10.24	HBE1	-6.25	IFI44L	8.61	HBE1	-9.09
LAG3	7.48	MARCHF2	-2.70	LAG3	7.24	TREML3	-4.17
IFI44	7.10	TREML3P	-2.63	GZMH	7.01	RUNDC3A	-3.70
GZMH	6.33	KIAA1324	-2.63	IFI44	6.13	TMEM158	-3.57
IFIT1	6.14	BEX1	-2.56	IFIT1	5.53	GYPA	-3.33
PPP2R2B	5.83	HS.545426	-2.50	IGJ	5.34	MARCHF2	-3.03
MCOLN2	5.12	SMIM5	-2.38	GBP5	5.19	SLC6A10P	-3.03
OAS3	5.07	FLCN	-2.38	PPP2R2B	4.98	BEX1	-2.94
GBP5	4.73	DPM2	-2.33	MCOLN2	4.79	TCP11L2	-2.94
JCHAIN	4.70	CABP5	-2.33	FGFBP2	4.52	KEL	-2.94

Supplementary Table 5: Top 10 upregulated and downregulated shared and unique genes identified in the discovery sets of infants with symptomatic or asymptomatic congenital cCMV infection.

Symptomatic and Asymptomatic cCMV shared gene List (2,160)		Unique Symptomatic cCMV gene List (432)				Unique Asymptomatic cCMV gene List (1,164)			
Top 10 upregulated	Top 10 downregulated	Top 10 upregulated	Fold change	Top 10 downregulated	Fold change	Top 10 upregulated	Fold change	Top 10 downregulated	Fold change
IFI44L	HBE1	CCZ1B	4.24	KIAA1324	-2.63	ITGB1BP1	3.30	RUNDC3A	-3.70
LAG3	MARCHF2	OTOF	3.67	SH3BGRL2	-2.13	CLECL1	2.86	TMEM158	-3.57
IFI44	TREML3	KIR2DL1	2.64	TREML1	-2.08	IDO1	2.37	GYPA	-3.33
GZMH	BEX1	ANKFY1	2.48	TNNC2	-1.96	DNTT	2.36	SLC6A10P	-3.03
IFIT1	HS.545426	HS.553301	2.24	TREM1	-1.92	TNFRSF13B	2.17	TCP11L2	-2.94
PPP2R2B	SMIM5	TRGV2	2.23	LY6G6F	-1.89	HRK	2.16	FLCN	-2.86
MCOLN2	FLCN	TMEM191A	2.16	RN5S9	-1.89	AL845472.2	2.13	TNS1	-2.86
OAS3	DPM2	MBTD1	2.16	VWF	-1.89	LOC650919	2.08	LOC732134	-2.78
GBP5	CABP5	SNRPGP5	2.12	ICAM4	-1.82	TSPAN13	2.00	SESN3	-2.70
IGJ	BPGM	MTE	2.12	GPR177	-1.82	IGKV5-2	1.99	HEMGN	-2.70

Supplementary Table 6: Comparison of differentially expressed genes amongst modular groups in symptomatic and asymptomatic congenital CMV infection

			Symptomatic cCMV		Asymptomatic cCMV		
Module	Function	Genes in Module	Differentially Expressed Genes (n)	Differentially Expressed Genes (%)	Differentially Expressed Genes (n)	Differentially Expressed Genes (%)	p-value
M1.2	Interferon	36	30	83%	30	83%	>0.999
M3.4	Interferon	62	42	68%	44	71%	>0.999
M5.12	Interferon	63	41	65%	36	57%	0.655
M4.14	Monocytes	62	-10	-16%	-7	-11%	0.856
M5.15	Neutrophils	24	0	0%	1	4%	>0.999
M3.2	Inflammation	147	-38	-26%	-68	-46%	0.104
M4.2	Inflammation	50	-7	-14%	-13	-26%	0.684
M4.6	Inflammation	116	-22	-19%	-28	-24%	0.788
M4.13	Inflammation	82	-46	-56%	-55	-67%	0.555
M5.1	Inflammation	244	-39	-16%	-29	-12%	0.714
M5.7	Inflammation	139	-35	-25%	-35	-25%	>0.999
M4.1	T cells	68	-4	-6%	1	1%	0.864
M4.15	T cells	45	16	36%	21	47%	0.816
M3.6	Cytotoxic T cells	53	34	64%	38	72%	0.598
M4.10	B-cells	34	32	94%	32	94%	>0.999
M4.11	Plasma cells	20	11	55%	10	50%	>0.999

p-values are calculated using a Fisher's exact T-test adjusted for multiple comparisons by Benjamini-Hochberg multiple test correction. p values were considered significant when $p<0.05$ using two-tailed tests. Positive values represent the number and proportion of genes overexpressed within a specific module, whereas negative values represent the number and proportion of genes that are underexpressed within a module.

Supplementary Table 7: Demographic characteristics of infants with congenital cCMV infection with and without late-onset SNHL

	cCMV with late-onset SNHL (n=24)	cCMV without late-onset SNHL (n=28)	p-value
Age at enrollment (days)	16 [10-22]	17 [15-21]	0.52
Gestational age (weeks)	39 [38.3-40]	39 [38-40]	0.89
Sex, males, n (%)	13 (54%)	20 (71%)	0.25
Race, n,<br>White<br>Black or other	 21<br>3	 26<br>2	0.65
Disease classification n (%)<br>Symptomatic cCMV<br>Asymptomatic cCMV	 13 (54%)<br>11 (46%)	 16 (57%)<br>12 (43%)	> 0.99

Statistical comparisons were performed using the Kruskal-Wallis test for non-parametric continuous variables and data reported as median, 25%-75% interquartile ranges, whereas Fisher's exact test was used for the analyses of categorical data. p values were considered significant when $p<0.05$ using two-tailed tests.

Supplementary Table 8: Sixteen classifier genes that accurately discriminate between infants with cCMV infection who went onto developing late onset SNHL

PROBE_ID	SYMBOL	Function
ILMN_1779257	CD40	From TNF-alpha receptor family (TNFRSF5). innate immune response. Needed for microglia activation. Altered in hearing loss.
ILMN_1798187	MYST2	(KAT7) Hystone acetyl transferase
ILMN_3241104	LOC286135	Non-coding protein
ILMN_1736650	JMJD2A	(KDM4A) protein coding gene
ILMN_2061950	RABGAP1	Protein coding gene. Cell metabolism
ILMN_1679092	RAB9B	Membrane trafficking; innate immune response
ILMN_2338038	AK3L1	(AK4) ATP metabolism.
ILMN_1660179	MATR3	Innate immune responses
ILMN_1779370	ARHGEF9	Protein coding gene. Associated with intellectual disability.
ILMN_1718520	C10orf59	(RNLS). Associated with vascular/renal regulation
ILMN_3237703	LOC645431	No functions available
ILMN_1655654	MPDU1	Protein metabolism. Associated with glycosylation defects/ intellectual disability.
ILMN_1680193	PAXIP1	DNA metabolism
ILMN_2193817	CLEC4G	Protein coding gene. Associated with CNS diseases.
ILMN_1781431	GLCCI1	Glucocorticoid related
ILMN_1804451	LEO1	Protein metabolism

Supplementary Figure 1: Consort diagram of enrolled healthy controls and infants with congenital CMV infection.

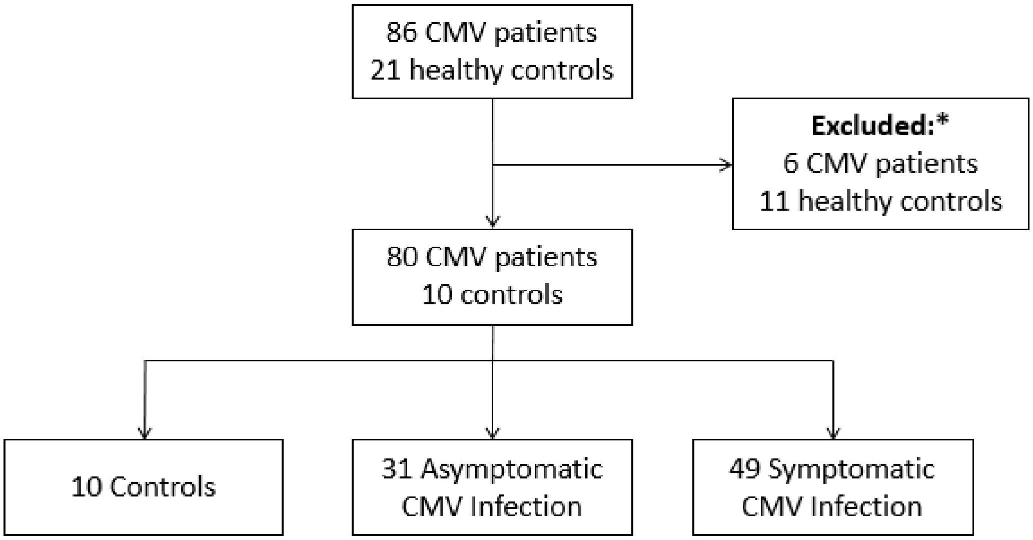

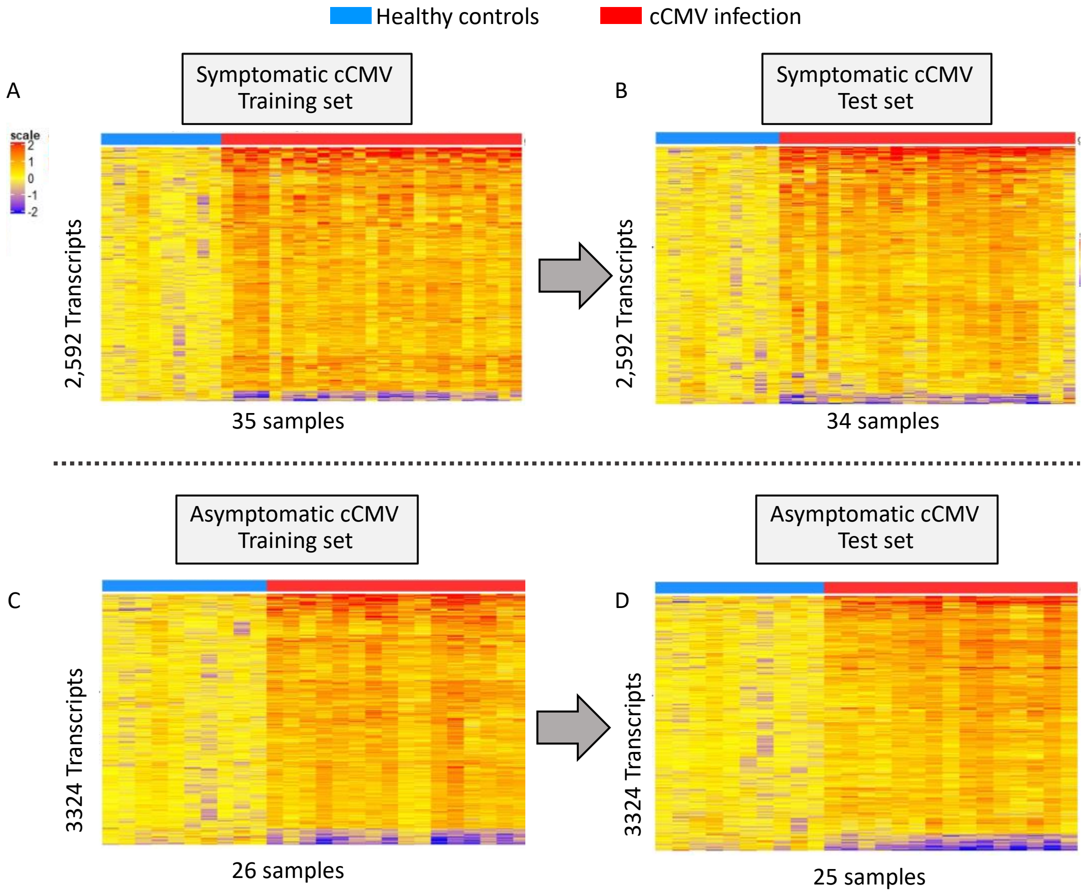

Supplementary Figure 2: Symptomatic and asymptomatic cCMV infection biosignatures are robust and reproducible. **(A)** Statistical group comparisons using linear models (LIMA) adjusted for age followed by Benjamini-Hochberg FDR <0.01 and ≥1.5 fold-change between infants with symptomatic cCMV infection (n=25) and healthy controls (n=10) identified 2,592 differentially expressed transcripts in the training set (symptomatic cCMV biosignature). **(B)** The transcriptional signature was validated in an independent test set of 24 infants with symptomatic cCMV and the same 10 healthy controls. Healthy controls are represented in blue and symptomatic cCMV patients in red. **(C)** A similar strategy was applied to derive and validate the asymptomatic cCMV biosignature using 16 cCMV infants in the training set and 10 healthy controls, which yielded 3,324 differentially expressed genes. **(D)** The asymptomatic cCMV biosignature was validated in an independent test set of 15 infants with asymptomatic cCMV infection and the same healthy controls. Transcripts are organized in a heatmap format where each row represents a transcript and each column a patient sample. Red color indicates overexpression and blue color underexpression of a transcript compared to the median expression of healthy controls (yellow).

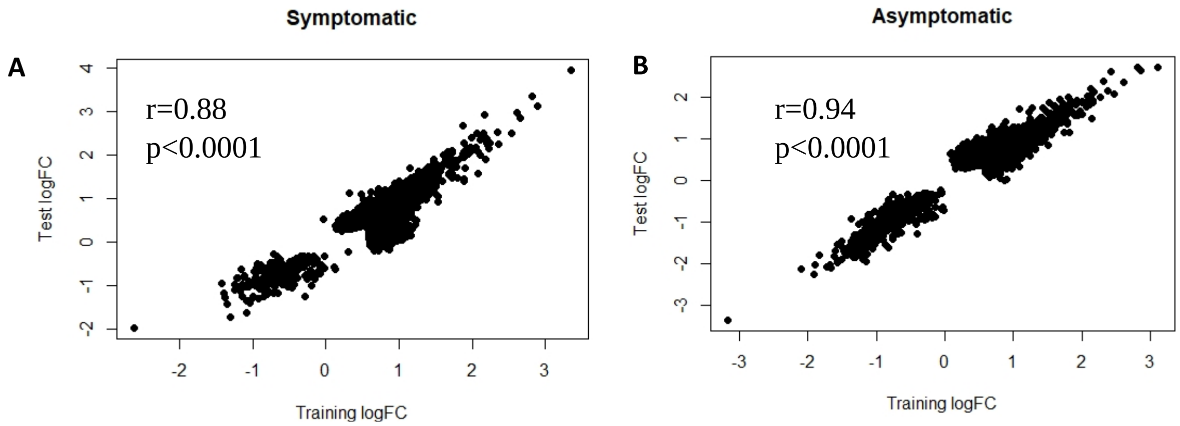

Supplemental Figure 3: Symptomatic and asymptomatic cCMV infection biosignatures are robust and reproducible**. (A)** Statistical group comparisons (Benjamini-Hochberg corrected FDR <0.01 and ≥1.5 fold-change) between infants with symptomatic cCMV infection (n=24) and healthy controls (n=10) identified 1,854 differentially expressed transcripts in the test set and 2,592 transcripts between 25 infants with symptomatic cCMV and healthy controls (training set; derivation). Spearman correlation between these two gene sets derived from infants with symptomatic cCMV infection showed high level of correlation. (B) Similarly, correlations between the gene sets derived from the test set (3,699 transcripts; n=15 asymptomatic cCMV infants/10 HC) and training set (3,324 transcripts; n=16 asymptomatic cCMV/10 HC) were high (r=0.94; p<0.0001).

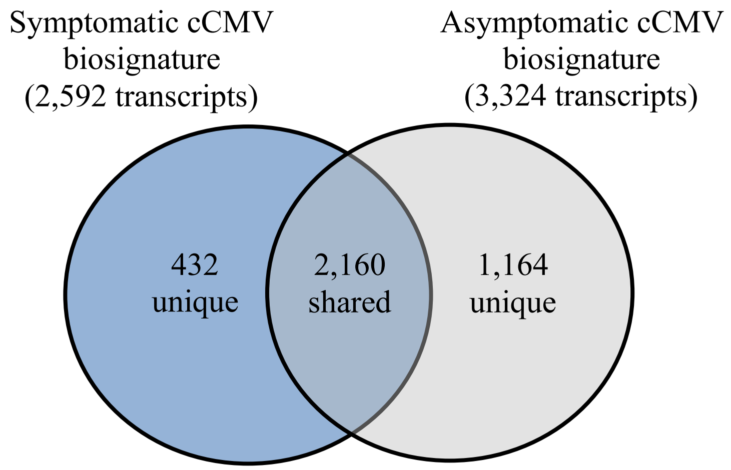

Supplementary Figure 4: Venn diagram: Overlay of symptomatic and asymptomatic cCMV biosignatures reveals both shared and unique transcripts. Overlay of symptomatic and asymptomatic cCMV biosignatures reveals both shared (2,160) and unique transcripts, 1,164 for infants with asymptomatic cCMV infection and 432 for those with symptomatic disease.

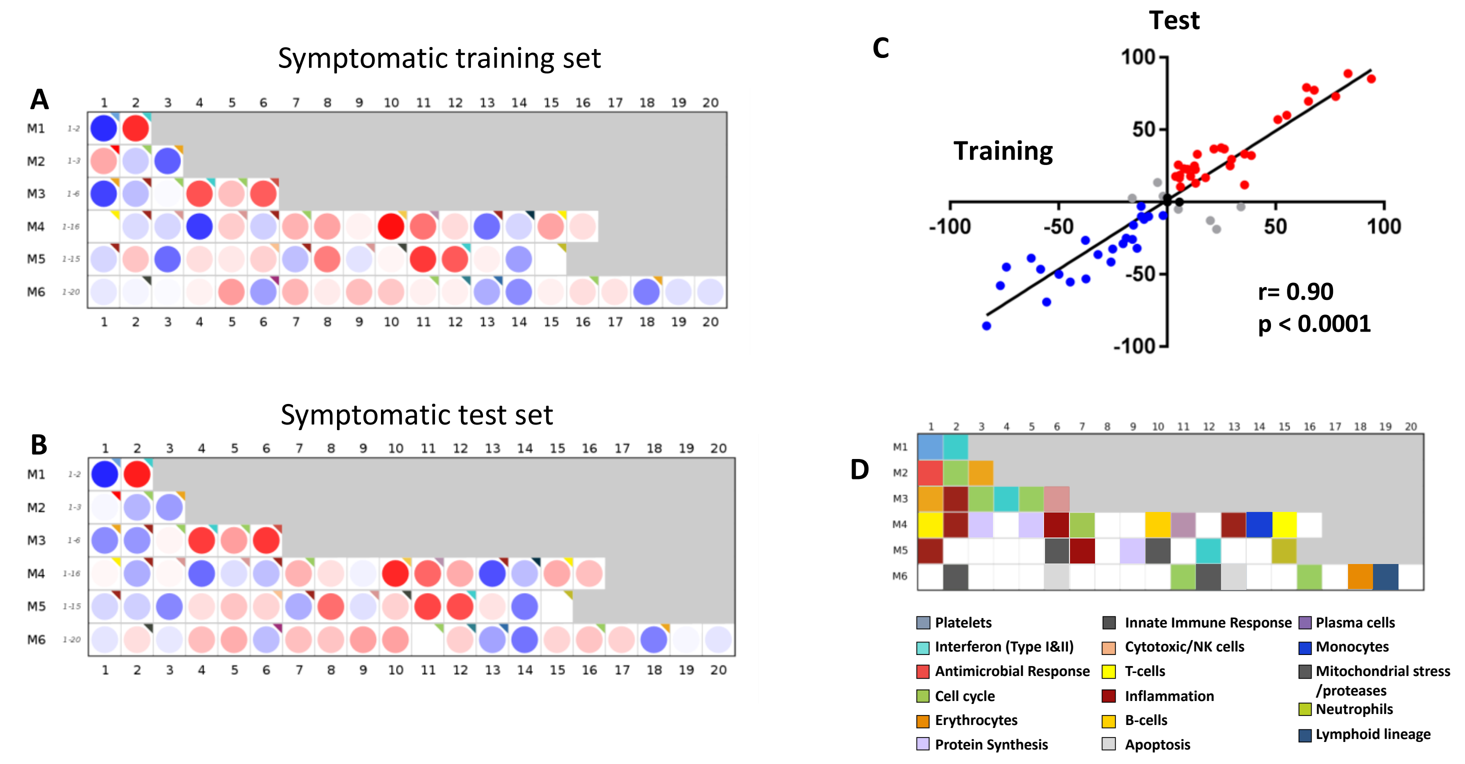

Supplementary Figure 5: Modular maps for the discovery and validation cohorts in symptomatic cCMV-infected infants. Modular maps were derived independently from the **(A)** training set (n=25) of infants with symptomatic cCMV infection and the **(B)** test set (n=24) and compared via a Spearman correlation **(C)**, demonstrating a high degree of similarity within the discovery and validation cohorts (r=0.90, p< 0.0001; Spearman correlation, multiple test corrections not applied). Each group was compared to 10 age-matched healthy controls. The intensity of the modules (dots) indicates the proportion of overexpressed (in red) or underexpressed (in blue) transcripts within each module. Blank dot indicates that <10% of the genes in the module were differentially expressed. **(D)** Modular maps legend.

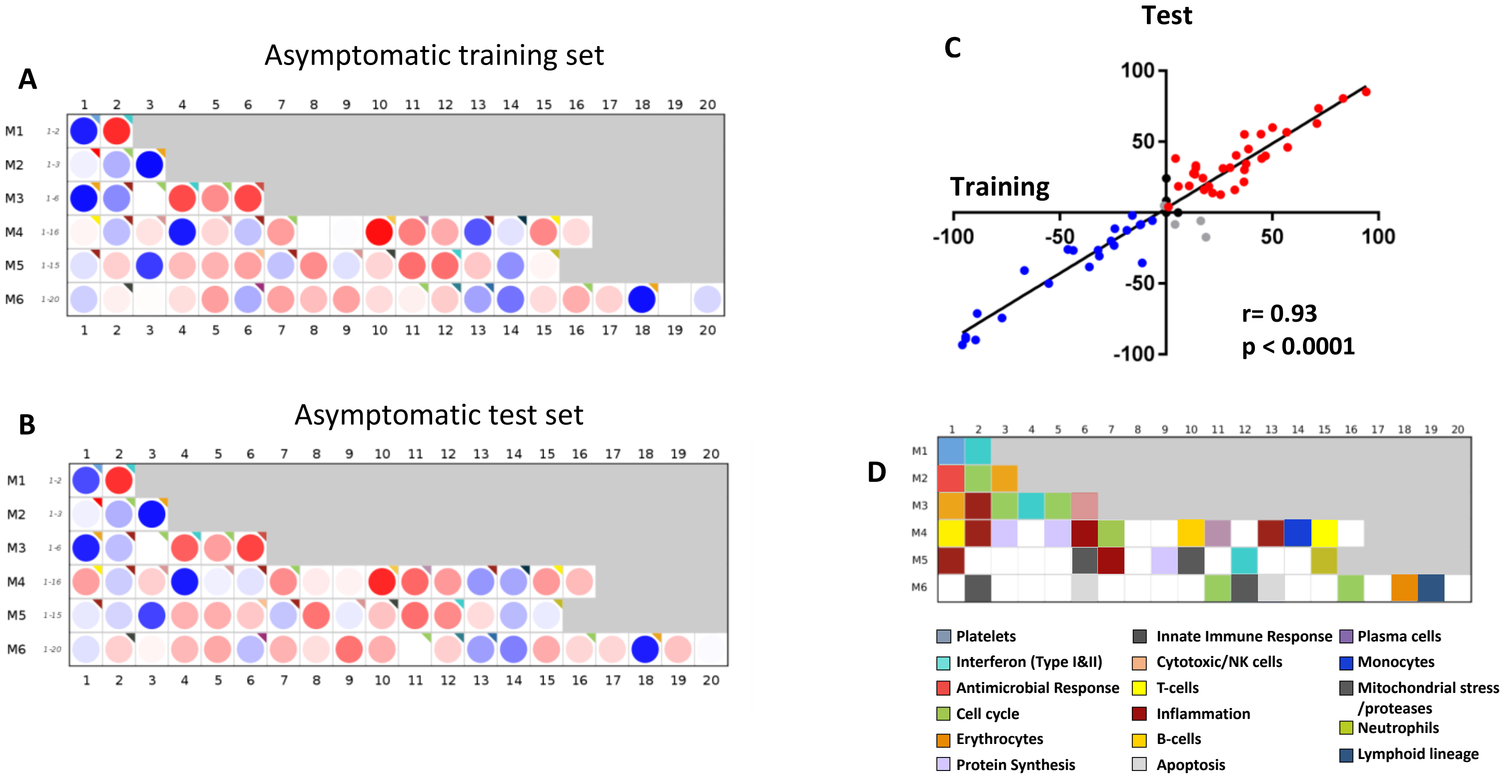

Supplementary Figure 6: Modular maps for the discovery and validation cohorts in infants with asymptomatic cCMV infection. Modular maps were derived independently from the **(A)** training set (n=16) of infants with asymptomatic cCMV infection and the **(B)** test set (n=15) and compared using Spearman correlation coefficient **(C)**, demonstrating a high degree of similarity within the discovery and validation cohorts (r=0.93, p< 0.0001; Spearman correlation, multiple test corrections not applied). Each group was compared to matched healthy controls. The intensity of the modules (dots) indicates the proportion of overexpressed (in red) or underexpressed (in blue) transcripts within each module. Blank dot indicates that <10% of the genes in the module were differentially expressed. **(D)** Modular maps legend.

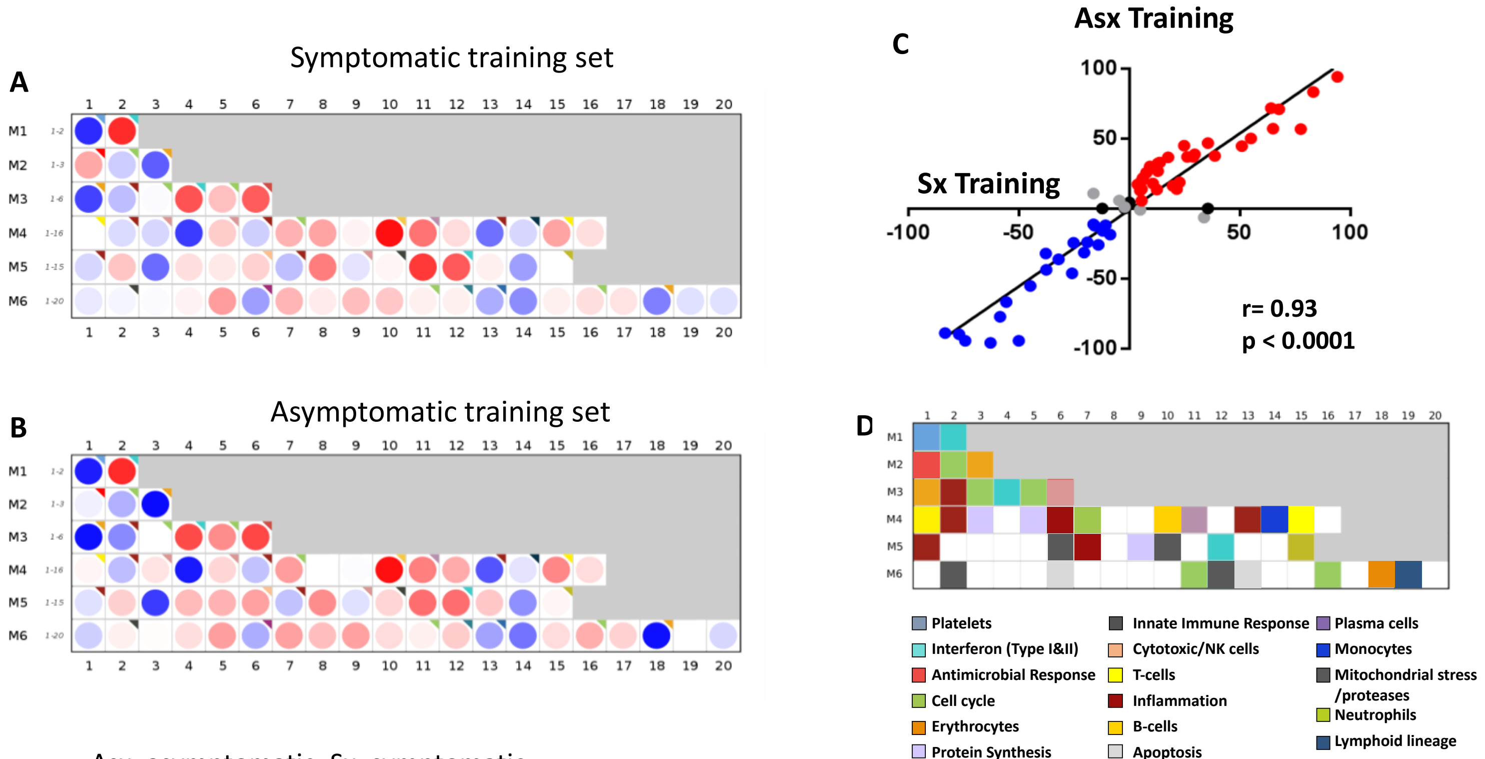

Supplementary Figure 7: Modular maps of the discovery cohorts for infants with asymptomatic and symptomatic cCMV infection. Modular maps derived from the **(A)** training set (n=25) of infants with symptomatic congenital CMV infection was compared with the **(B)** training set (n=16) of infants with asymptomatic cCMV infection using Spearman correlations coefficient **(C)**, demonstrating a high degree of similarity between both discovery cohorts (r=0.93, p< 0.0001; Spearman correlation, multiple test corrections not applied), further supporting results from unsupervised cluster analyses. Asx: asymptomatic, Sx: symptomatic. Each cohort was compared to matched healthy controls. The intensity of the modules (dots) indicates the proportion of overexpressed (in red) or underexpressed (in blue) transcripts within each module. Blank dot indicates that <10% of the genes in the module were differentially expressed. **(D)** Modular maps legend.

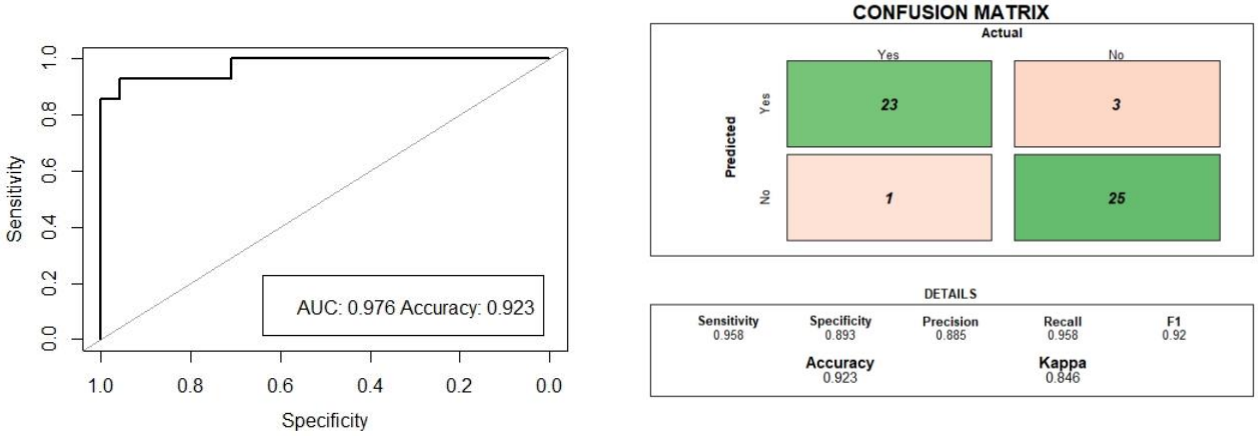

Supplementary Figure 8: Identification and validation of classifier genes for the development of sensorineural hearing loss (SNHL) in infants with either symptomatic or asymptomatic cCMV infection using Random Forest-Recursive Feature Elimination (RF-RFE). Within the cohort of infants with cCMV infection 24 infants developed SNHL at any time point during the follow-up period whereas 28 did not develop SNHL and had at least 900 days of follow-up. (A) Random Forest-Recursive Feature Elimination (RF-RFE) algorithm identified a 16-gene signature that classified cCMV infants who developed SNHL with 92.3% accuracy. (B) Performance of classification is shown using a confusion matrix.
