# Supplementary material for: Blood genome expression profiles in infants with congenital cytomegalovirus infection
Source: Nat Commun. 2020 Jul 15;11:3548. doi: 10.1038/s41467-020-17178-5 (PMC7363904; doi:10.1038/s41467-020-17178-5)
Supplement: Supplementary file 3 — Reporting Summary [file 41467_2020_17178_MOESM3_ESM.pdf]

nature research

Corresponding author(s): Asuncion Mejias

Last updated by author(s): May 26, 2020

Reporting Summary

Nature Research wishes to improve the reproducibility of the work that we publish. This form provides structure for consistency and transparency in reporting. For further information on Nature Research policies, see Authors & Referees and the Editorial Policy Checklist .

Statistics

For all statistical analyses, confirm that the following items are present in the figure legend, table legend, main text, or Methods section.

n/a	Confirmed	
☐	☒	The exact sample size (*n*) for each experimental group/condition, given as a discrete number and unit of measurement
☒	☐	A statement on whether measurements were taken from distinct samples or whether the same sample was measured repeatedly
☐	☒	The statistical test(s) used AND whether they are one- or two-sided<br>*Only common tests should be described solely by name; describe more complex techniques in the Methods section.*
☐	☒	A description of all covariates tested
☐	☒	A description of any assumptions or corrections, such as tests of normality and adjustment for multiple comparisons
☐	☒	A full description of the statistical parameters including central tendency (e.g. means) or other basic estimates (e.g. regression coefficient) AND variation (e.g. standard deviation) or associated estimates of uncertainty (e.g. confidence intervals)
☐	☒	For null hypothesis testing, the test statistic (e.g. *F*, *t*, *r*) with confidence intervals, effect sizes, degrees of freedom and *P* value noted<br>*Give P values as exact values whenever suitable.*
☒	☐	For Bayesian analysis, information on the choice of priors and Markov chain Monte Carlo settings
☐	☒	For hierarchical and complex designs, identification of the appropriate level for tests and full reporting of outcomes
☒	☐	Estimates of effect sizes (e.g. Cohen's *d*, Pearson's *r*), indicating how they were calculated

Our web collection on statistics for biologists contains articles on many of the points above.

Software and code

Policy information about availability of computer code

Data collection	No specific software was utilized for data collection.
Data analysis	Microarray data analysis was performed using JMP genomics and R software packages for analytic purposes. Statistical analyses were performed using GraphPad Prism V6

For manuscripts utilizing custom algorithms or software that are central to the research but not yet described in published literature, software must be made available to editors/reviewers. We strongly encourage code deposition in a community repository (e.g. GitHub). See the Nature Research guidelines for submitting code & software for further information.

Data

Policy information about availability of data

All manuscripts must include a data availability statement. This statement should provide the following information, where applicable:

- Accession codes, unique identifiers, or web links for publicly available datasets
- A list of figures that have associated raw data
- A description of any restrictions on data availability

Raw data for the microarray analysis was deposited in the NCBI Gene Expression Omnibus GEO accession number GSE108211. All figures have been generated after analyses of the baseline raw data.

Field-specific reporting

Please select the one below that is the best fit for your research. If you are not sure, read the appropriate sections before making your selection.

Life sciences study design

All studies must disclose on these points even when the disclosure is negative.

Sample size	Best practices for transcriptome analyses recommend cohorts of ~ 20 patients in the discovery phase (training test) and an independent cohort of 15-20 patients for validation purposes (test set), which is what was done. Patients included in the discovery cohorts were selected randomly. The number of patients enrolled included those available for study purposes, and robust statistical analyses were performed to ensure reproducibility of data.
Data exclusions	Patients were excluded if the RNA quality for transcriptional analysis was suboptimal by either quantity or quality. These quality control analyses were performed at the onset of study design, thereby resulting in a total population of 80 participants and 10 healthy controls (initially enrolled 86 patients and 21 healthy controls).
Replication	All data underwent replication to demonstrate reproducibility. Notably, all transcriptional analyses underwent generation of the discovery and validation cohorts. These groups were randomly selected, and biosignatures of both "symptomatic" and "asymptomatic" congenital CMV had highly reproducible results. This is done in a single measure (one discovery cohort, one test cohort, for each of the symptomatic and asymptomatic biosignatures). Importantly, we performed additional methods of reproducibility (such as spearman correlations) to ensure reproducibility of the discovery and test cohorts. Additionally, a similar approach was taken with modular analyses to ensure the reproducibility of the symptomatic and asymptomatic biosignatures. Modular maps were also generated for the discovery and validation cohorts of patients with symptomatic and asymptomatic cCMV infection and results also correlated to further demonstrate the reproducibility of the biosignatures within the dataset.
Randomization	As this is not an interventional study, there was no randomization with respect to patient enrollment. However, for data analyses and when identifying discovery and test cohorts, patients in the symptomatic and asymptomatic cohorts were included in the training (discovery) or test (validation) sets randomly.
Blinding	To demonstrate the robust nature of our analyses, the test cohort was evaluated using an unsupervised analysis, and as such the program is "blinded" to the condition of the patients (which demonstrated highly reproducible results).

Reporting for specific materials, systems and methods

We require information from authors about some types of materials, experimental systems and methods used in many studies. Here, indicate whether each material, system or method listed is relevant to your study. If you are not sure if a list item applies to your research, read the appropriate section before selecting a response.

Materials & experimental systems

n/a	Involved in the study	
☒	☐	Antibodies
☒	☐	Eukaryotic cell lines
☒	☐	Palaeontology
☒	☐	Animals and other organisms
☐	☒	Human research participants
☐	☒	Clinical data

Methods

n/a	Involved in the study	
☐	☒	ChIP-seq
☒	☐	Flow cytometry
☒	☐	MRI-based neuroimaging

Human research participants

Policy information about studies involving human research participants

Population characteristics	Relevant population characteristics considered for this study included age at diagnosis, age at sample collection, gestational age, sex, and race. These characteristics were compared to ensure similar baseline characteristics of our healthy controls and infants with congenital CMV infection (both symptomatic and asymptomatic). All variables, with exception patient gestational age, were similar (ex. did not reach statistical difference) among healthy controls, symptomatic, and asymptomatic congenital CMV infants. While gestational age (GA) did reach a statistical difference, the median GA of healthy controls was 38 weeks, whereas the median GA of congenital CMV infants (asymptomatic and symptomatic) was 39 weeks. Birth at > 37 weeks is often considered a "term" birth, and thus this finding is likely one of statistical significance though not of clinical relevance (and thus supporting the similarities of the cohorts).
Recruitment	All patients identified with congenital CMV infection (based on detection of CMV by PCR of culture from blood, urine, or saliva within the first 21 days of age) were approached for enrollment in this study at Parkland Memorial Hospital (Dallas) or Nationwide Children's Hospital (Columbus). Several factors influenced screening. Identification of patients with signs or symptoms of congenital CMV infection (ex. microcephaly, thrombocytopenia, referred hearing screen) would prompt screening. Importantly, this would only identify symptomatic infants. The infants in our asymptomatic cohort were identified through a concurrent study (CHIMES study) - a study that undertook universal screening for congenital CMV infection to better define the impact of congenital CMV infection. Thus, recruitment was not biased to those only with clinical signs or symptoms of infection. No other biases were present in our recruitment efforts. We do not believe any external biases are present that would have otherwise altered our results.
Ethics oversight	This study was approved by the institutional review boards at Nationwide Children's Hospital (Columbus, OH) and the University of Texas Southwestern Medical Center (Dallas, TX).

Note that full information on the approval of the study protocol must also be provided in the manuscript.

Clinical data

Policy information about clinical studies

All manuscripts should comply with the ICMJEguidelines for publication of clinical research and a completedCONSORT checklist must be included with all submissions.

Clinical trial registration	n/a
Study protocol	Protocols were included in the IRB at UT Southwestern Medical Center Dallas and Nationwide Children's Hospital, Columbus, OH
Data collection	Data was collected upon enrollment and after completion of follow-up visits at UT Southwestern Medical Center and at Nationwide Children's Hospital (from 2007 through 2013.
Outcomes	The primary outcome was to evaluate for differences in whole blood genome expression profiles between infants with symptomatic and asymptomatic cCMV infection. Symptomatic CMV infection was defined as an clinical, laboratory, neuroradiologic, and audiologic abnormality at diagnosis (within the first 21 days of age) consistent with congenital CMV infection. All other infants were considered as having asymptomatic congenital CMV infection. Multiple methods were applied to evaluate for differences at the transcriptional, modular, and global level of blood genome expression profiles, though we did not identify differences between symtpomatic and asymptomatic congenital CMV infection. Our secondary outcome was to evaluate for a biosignature predictive of sensorineural hearing loss. Infants with congenital CMV infection and without hearing loss at birth, who had at least 900 days of follow up, were included in evaluation. With this, we were able to identify a 16-gene set, present at diagnosis, that was 92% accurate in identifying those infants who would develop sensorineural hearing loss.

ChIP-seq

Data deposition

- [x] Confirm that both raw and final processed data have been deposited in a public database such as GEO.
- [ ] Confirm that you have deposited or provided access to graph files (e.g. BED files) for the called peaks.

Data access links<br>*May remain private before publication.*	https://www.ncbi.nlm.nih.gov/geo/query/acc.cgi<br>(the accession to the dataset is currently private and will be released upon manuscript acceptance or on December 2020, whatever happens first)
Files in database submission	Clinical information is provided in tables, raw and processed genomic information is deposited in GEO-- se above--
Genome browser session<br>(e.g. UCSC)	https://www.ncbi.nlm.nih.gov/geo/query/acc.cgi

Methodology

Replicates	no replicates of individual samples were performed in this study. Reproducibility of our data is discussed above

Sequencing depth	sequencing was not performed in this study
Antibodies	no antibodies were used in our study
Peak calling parameters	Read mapping was not performed in this study. Data quality is reported as below.
Data quality	- The MagMAX RNA Isolation and BioSprint96 Robot SOP.EXT3.1. was used to extract 195 samples<br>- Sample quality was assessed using the bioanalyzer (16s/18s ratio), and quantity was evaluated by assessing the “rna integrity number” (RIN) using the nanodrop.<br>- We assessed the quality and quantity of total RNA (tRNA) and globin reduced RNA (grRNA).<br>- Briefly, as a first step, the RIN average of the samples was 8.3, and the RNA yield average was 5017ng. The total RNA was then globin reduced using the GlobinClear Human- SOP.GLOB4.v2. The RIN average of the samples passed were 7.2 and gRNA yield average is 1707ng. Samples with a RIN below 7 or insufficient quantity were not hybridized.<br>- For amplification the average cRNA yield is 21,748ng (cut off of 10,000). Some samples had goblin peak profiles. All samples but one were hybridized successfully with passing QC parameters. Data QC included:<br>o The detected genes at p<0.05 and p<0.01 that fell in range between Upper and Lower Cutoff limits (UCL and LCL)<br>- Detected genes at p<0.01 (average UCL-LCL: 10945.46 [13275.91-8615.00]<br>- Detected genes at p<0.05: 13687.87 [16084.15-11291.59]<br>o The Signal Average and Signal P95 fell in range between UCL and LCL<br>- Signal average: 170.61 [279.12-62.10]<br>- Signal P95: 602.18 [1055.56-148.80]<br>o The Biotin and Housekeeping fell in range between UCL and LCL<br>- Biotin: 11868.78 [16953.61-6783.95]<br>- Housekeeping: 6113.10 [10053.25-2172.96]<br>o Negative background fell in range between UCL and LCL: 99.08 [118.27-79.89]
Software	Microarray data analysis was performed using JMP genomics, and R software packages for analytic purposes.
